# Editorial: Lexical Tone Perception in Infants and Young Children: Empirical Studies and Theoretical Perspectives

**DOI:** 10.3389/fpsyg.2019.01195

**Published:** 2019-05-22

**Authors:** Leher Singh, Denis Burnham, Jessica Hay, Liquan Liu, Karen Mattock

**Affiliations:** ^1^Department of Psychology, National University of Singapore, Singapore, Singapore; ^2^MARCS Institute for Brain, Behaviour & Development, Western Sydney University, Penrith, NSW, Australia; ^3^Department of Psychology, The University of Tennessee, Knoxville, TN, United States; ^4^Department of Psychology, Western Sydney University, Penrith, NSW, Australia

**Keywords:** language acquision, psycholinguistic abilities, mandarin Chinese, lexical tones, speech perception

Traditional theories of language development and speech processing have been derived from psycholinguistic research that has primarily focused on a particular subset of language types. Specifically, Romance and Germanic languages (e.g., English, French, German) have, until recently, received more attention than other types of languages, such as Chinese languages. This has led to selective emphasis on consonants and vowels—the phonological building blocks of European languages—in theories of language, to the exclusion of other phonological building blocks, such lexical tone. Like consonants and vowels, variations in tones determine lexical meaning, but unlike consonants and vowels, lexical tones are based on pitch variations. Lexical tone is pervasive; it is used in at least half of the world's languages (Maddieson, 2013), including most Asian and some African, Central American, and European languages. This Research Topic brings together a collection of recent empirical research on the processing and representation of lexical tones across the lifespan with an emphasis on advancing knowledge on how tone systems are acquired and enriching current theories of language processing and development. The articles focus on various aspects of tones: its early perception, influences on word learning, the acquisition of new tone systems, and tone production. One set of articles report on tone perception at the earliest stage of development, in infants learning either tone or non-tone languages. Tsao and Chen et al. demonstrate that, in contrast to traditional accounts of perceptual narrowing for consonants and vowels, infants' sensitivity to Mandarin lexical tone, as well as pitch, improves over the first year of life in both native and non-native learners. Götz et al. report a U-shaped developmental trajectory for Cantonese tone perception and illustrate how the choice of methodological approaches can influence findings on infants' tone sensitivity. Fan et al. demonstrate that sensitivity to less well-studied properties of tone languages, such as neutral tone, may develop after the first year of life. Cheng and Lee investigate native tone discrimination in an electrophysiological study during the second year of life and report effects of stimulus salience on infants' neural response to tones. In a complementary set of studies focused on tone sensitivity in word learning, Burnham et al. demonstrate that infants bind tones to newly-learned words if they are learning a tone language, either monolingually or bilingually, although it was also found that object-word binding was influenced by the properties of individual tones. Shi et al. also demonstrate effects of stimulus properties on tone-object binding in native learners of Mandarin. Liu and Kager chart a developmental trajectory over the second year of life in which infants narrow in their interpretation of non-native tones. Choi et al. investigate how learning a tone language can influence uptake of other suprasegmental properties of language, such as stress, and demonstrate that native tone sensitivity in children can facilitate stress sensitivity when learning a stress-based language. Finally, two studies focus on sensitivity to pitch in a sub-class of tone languages: pitch accent languages. In a study on Japanese children's abilities to recognize words they know, Ota et al. demonstrate a limited sensitivity to native pitch contrasts in toddlers. In contrast, Ramachers et al. demonstrate comparatively strong sensitivity to pitch in native and non-native speakers of a different pitch accent system (Limburgian) when learning new words. Several studies focus on learning new tone systems. In a training study with schoolaged children, Kasisopa et al. demonstrate that tone language experience increases children's abilities to learn new tone contrasts. Poltrock et al. show similar advantages of tone experience in learning new tone systems in adults. In an electrophysiological study, Liu et al. demonstrate order effects in adults' neural responses to new tones, discussing implications for learning tone languages as an adult. Finally, Hannah et al.'s work suggests that extralinguistic cues, such as facial expression, can support adults' learning of new tone systems. In the first of three studies investigating tone production, Rattanasone et al. report the results of a study demonstrating kindergartners' asynchronous mastery of tones —i.e., delayed acquisition of tone sandhi forms relative to base forms. In a study examining a corpus of adult tone production, Han et al. demonstrate that mothers produce tones in a distinct manner when speaking to infants: tone differences are emphasized more when speaking to infants than to adults. Finally, combining perception and production of tones, Wong et al. report asynchronous development of tone perception and tone production in children. The Research Topic also includes a series of Opinion pieces and Commentaries addressing the broader relevance of tone and pitch to the study of language acquisition. Curtin and Werker discuss ways in which tone can be integrated into their model of infant language development (PRIMIR). Best discusses the phonological status of lexical tones and considers how recent empirical research on tone perception bears on this question. Kager focuses on how language learners distinguish lexical tones from other sources of pitch variation (e.g., affective and pragmatic) that also inform language comprehension. Finally, Antoniou and Chin unite evidence of tone sensitivity from children and adults and discuss how these areas of research can be mutually informative. Over the past decade, psycholinguistic studies of lexical tone acquisition have begun to burgeon. This collection of empirical studies and opinion pieces provides a state of-the-art panoply of the psycholinguistic study of lexical tones, and attest to its relevance to language acquisition research. The articles in this Research Topic will help address past biases toward European non-tone languages, and will contribute to an expanding narrative of speech perception, speech production, and language acquisition that draws from a greater diversity of languages. Importantly, these studies underline the scientific promise of psycholinguistic research on tone languages; the research questions raised by the study of lexical tone are new and complement those typically applied to more widely studied languages and populations. Studies on lexical tone will continue to enrich psycholinguistic research in language acquisition and processing in a way that brings us closer to universal principles of language development.

## Author Contributions

LS, DB, JH, and LL drafted and edited the editorial. KM helped to draft and edit the proposal for this research topic.

### Conflict of Interest Statement

The authors declare that the research was conducted in the absence of any commercial or financial relationships that could be construed as a potential conflict of interest.
